# Lateral Motor Column specific expression of Sonic Hedgehog contributes to maintenance and scaling of pMN progenitor cell populations during oligodendrogenesis

**DOI:** 10.21203/rs.3.rs-4249282/v1

**Published:** 2024-05-13

**Authors:** Lev Starikov, Miruna Ghinia-Tegla, Andreas H. Kottmann

**Affiliations:** 1 City University of New York School of Medicine (CSOM), Dept. of Molecular, Cellular and Biomedical Sciences, New York City, NY 10031, USA.; 2 City University of New York Graduate Center, Molecular, Cellular, and Developmental Biology Subprogram, New York City, NY 10016, USA.; 3 City College of New York, Dept. of Biology, New York City, NY 10031, USA.; 4 City University of New York Graduate Center, Neuroscience Subprogram, New York City, NY 10016, USA3

**Keywords:** Sonic hedgehog signaling, motor neurons, pMN domain, oligodendrocyte precursor, spinal cord sculpting

## Abstract

Motor neurons (MNs) and oligodendrocyte precursor cells (OPCs) emerge sequentially from the pMN precursor domain during spinal cord development. MNs diversify into muscle specific subtypes and settle in stereotypic locations in the ventral horns. In contrast, OPCs are mobile and appear to evenly populate the parenchyma. Whether earlier born MNs influence OPC production is controversial. We found that Sonic Hedgehog signaling emanating from nascent MNs of the lateral motor column is critical for maintaining a larger and more yielding pMN domain at limb levels compared to trunk levels during OPC production. Reduced Shh signaling resulted in unrecoverable diminishment of pMN domain based OPC production leaving the spinal cord impoverished of OPC. Our results suggest that production of OPC at limb levels is contingent on completion of MN production.

## Introduction

The constituent parts of the central nervous systems (CNS) of most organisms develop sequentially and proportionately. However, the mechanisms by which proportionality emerges during development remain incompletely understood (reviewed in^1^. Development relies on the spatially patterned diversification of precursor cells which is enabled by positional information imparted onto those precursor cells by gradients of morphogens emanating from fixpoints within the developing embryo. For example, precursor cells located in the pMN domain, which stretches along the entire rostral to caudal extent of the developing neural tube, produce first motor neurons (MNs) and then oligodendrocyte precursor cells (OPCs)^2,3 4–9^. Nascent MNs diversify rapidly into spinal level specific subtypes according to the rostro-caudal positional information which they inherit from their precursor cells in form of the specific expression of *Hox* family transcription factors^10–12^. As a result, the mature spinal cord of tetrapods develops prominent enlargements at segmental levels that provide neuronal control of limbs. The larger spinal cord at brachial (forelimb) and lumbar (hindlimb) levels compared to thoracic (trunk) levels is the result of the presence of a greater number of MNs that subserve limb musculature, and, correspondingly, larger numbers of glia cells^13–16^. In contrast to MNs, OPCs remain mitotically active, highly mobile and tile the parenchyma which is thought to result in an appropriate density of oligodendrocytes throughout the neuroaxis and questioning the need for OPCs to retain positional information about their rostro-caudal origin of production^17–19^.

Many studies have implicated the morphogen Sonic Hedgehog (Shh) in regulating both, MN and OPC production in the pMN domain at multiple developmental stages^5,20–22^. First, graded Shh signaling originating from the notochord induces distinct transcriptional programs in overlying neural ectoderm cells in a concentration-dependent manner that leads to the establishment of molecularly distinct precursor domains along the ventral to dorsal axis of the developing neural tube^23–25^. The transcription factors activated by Shh are responsible for determining the cell fates in the derivatives of these precursor domains and include the basic helix-loop-helix (bHLH) transcription factor Olig2, which defines the pMN domain^26,27 28^.Subsequently, persistent Shh signaling originating from the medial floorplate (MFP) located at the ventral midline of the developing neural tube is critical for maintaining the identities of these precursor domains throughout neurogenesis^29^. The neural tube expands rapidly during neurogenesis and Shh signaling strength measured by expression levels of Shh target genes declines progressively within the ventral precursor domains^30,31^. Curiously, however, the beginning of oligodendrogenesis is marked by increased Shh signaling strength within the pMN domain^32^. The increase in Shh signaling was suggested to be critical for determining the time of cessation of MN production and onset of oligodendrogenesis^33^. Since the Shh gradient emanating from the MFP displays a constant decay length and does not scale with rapid growth^34^, its influence on pMN domain activity must likely wane during neurogenesis. Accordingly, multiple adaptation mechanisms have been identified that underlie the increase in Shh signaling strength in the pMN domain at the time of initiation of OPC production. One such mechanism is the accumulative storage of Shh in the extracellular matrix followed by Sulfatase 1 dependent release which subsequently leads to a greater concentration of Shh than could be achieved by continuous production and diffusion^22,32,35,36^. Another mechanism is the timed and graded expression of the Shh co-receptor Boc, which increases the efficacy of Shh signaling on Shh target cells and is required for pMN progenitor maintenance^37^. A further contributing mechanism might be the sequential production of Shh by previously specified ventricular zone derivatives (VZD).

Prominent sources of VZD produced Shh (VZD_Shh_) are the lateral floorplate (LFP_Shh_), which is constituted by cells emigrating from the most ventral and MFP abutting P3 precursor domain^36,38,39^, and nascent MNs which emerge from the pMN domain (MN_Shh_)^40–42^. The potential physiological significance of VZD_Shh_ was recently highlighted by the finding that the ablation of Shh by Olig2-Cre causes a failure of lateral motor column specification^42^ and ventral oligodendrogenesis^43^. However, the interpretation of these findings in regard of the specific contributions of different sources of Shh in the ventral spinal cord towards MN specification and OPC generation is made difficult by the transient expression of Olig2 in cells of the MFP and P3 domain in addition to precursor cells in the pMN domain and their descendants^27,44^. To overcome these technical difficulties, we produced a series of mouse lines with different degrees and tissue selectivity of conditional Shh gene ablation from the MFP, LFP, and MNs during spinal cord development. We observed that early neural tube patterning and MN development proceeded successfully in the absence of VZD_Shh_ sources, but that the pMN domain became exhausted of Olig2 expressing progenitor cells during neurogenesis prior to oligodendrogenesis. Our experiments revealed that LFP_Shh_ is needed for pMN progenitor maintenance throughout the spinal neuraxis. In contrast, MN_Shh_ expression is critical for maintaining a larger pMN progenitor cell population at brachial levels compared to thoracic segments during OPC production. Our data suggests that motor column specific expression of Shh breaks the otherwise monotonous expression of Shh at the ventral midline along the rostro-caudal axis, provides a mechanism by which oligodendrogenesis can only commence when lateral motor column formation completes and contributes to sculpting the tetrapod spinal cord.

## Results

### Expression and conditional ablation of Shh in the ventral spinal cord during MN- and OPC- production.

**Fig. 1:**
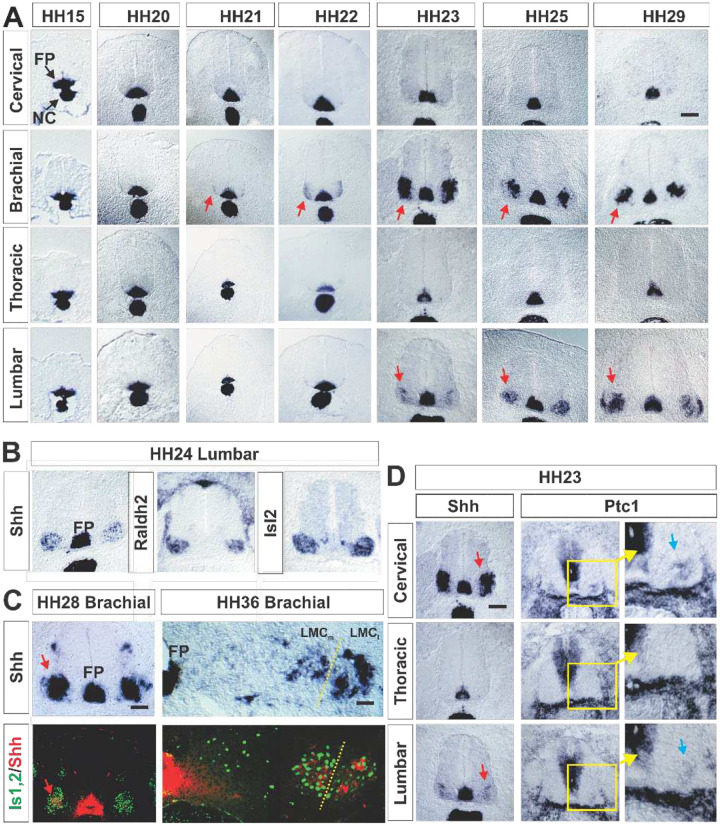
Shh expression in MNs. (**A**) In situ mRNA hybridization for Sonic Hedgehog (Shh) in developing chick neural tube revealing prominent expression in ventral horns at limb levels in presumptive motor neurons (MNs) at Hamilton/Henderson (HH) stage 21 at brachial and at HH23 at lumbar levels. Arrows point to nascent MNs in the lateral horns. FP: floor plate; NC: Notochord. (**B**) in situ mRNA hybridization on adjacent sections demonstrating overlap of the expression domains of the pan MN marker Isl2, retinolaldehyddehydrogenase 2 (Raldh2) and Shh. (**C**) mRNA in situ hybridization for Shh and immunohistochemical co-staining for Shh protein (red arrows) and the pan MN marker Isl2 on adjacent sections revealing Shh expression by MNs of the medial and lateral halves of the lateral motor column (LMCm and LMCl, resp.) at HH28 and HH36. (**D**) mRNA in situ hybridization for Shh and Ptc1 on adjacent sections. Red arrows point to Shh expressing MNs, blue arrows point to Ptc1 expressing MNs Scale bars: 100 μm.

In order to assess the contributions of VZD_Shh_ to pMN domain activity we first expanded on previous observations of Shh expression during spinal cord development in chick from Hamburger – Hamilton (HH,^45^) stages 15 (onset of MN production^46^) to HH29 (MN production complete and columnar organization of MN established^47,48^). At each stage we found prominent expression of Shh in the notochord and floorplate (Fig. 1A). In addition, we observed expression of Shh by cells in the developing ventral horns of the spinal cord at brachial levels from stage HH21 and lumbar levels from stage HH23 onwards (red arrows in Fig. 1A), but not at cervical or thoracic levels.

Retinoic acid can induce Shh expression^49–52^. It was previously found that expression of the rate limiting enzyme in the production of Retinoic acid, Retinolaldehyd dehydrogenase 2 (Raldh2), is expressed by MNs of the LMC beginning at HH19 at brachial and at HH21 at lumbar levels^53^. We found that the domain of Shh expression in the lateral horns overlaps with the expression area of Raldh2 and the pan-MN marker Isl2 at HH24 (Fig. 1B) consistent with the possibility that retinoic acid production in LMC MNs induces Shh expression in the lateral horns.

Consistent with the mRNA expression data, we found Shh protein expression in MNs of the LMC at HH28 (Fig. 1C, **red arrows**). Selective expression of Shh in MNs of the LMC is maintained until after the phase of MN cell death at HH36. At that time, Shh becomes expressed in a complex, MN-pool or -sub pool specific pattern (Fig. 1C, **red arrows**). Shh signaling boosts basal transcription levels of the Shh receptor Patched (Ptc1)^54,55^. Consistent with the emergence of signaling competent Shh from MNs, we found Ptc1 mRNA expression in the ventral horns at brachial and lumbar levels, but not thoracic levels, in addition to strong Ptc1 expression along the midline at HH23 (Fig 1D, **blue arrows in enlargements**). Curiously, however, despite starkly different mRNA expression levels of Shh in the ventral horns at brachial (high) and lumbar (low) at stage HH23 (**red arrows**, Fig. 1D), Ptc1 mRNA expression levels were similar at both sites (**blue arrows in enlargements** Fig. 1D). This observation pointed to the possibility that MN produced Shh might mainly signal to cells outside of the lateral horns.

We next investigated Shh expression in the developing ventral spinal cord in mice using a gene expression tracer and conditional loss of function allele of Shh (Shh^C^), in which a bi-cistronic mRNA is transcribed from the un-recombined Shh locus that encodes Shh and nuclear targeted LacZ^56^; Jackson Lab/MGI:7465089, Fig. 2A). Homozygous Shh^C/C^ mice are born with normal mendelian frequency, thrive, are fertile and are morphologically inconspicuous^56^.

**Fig. 2:**
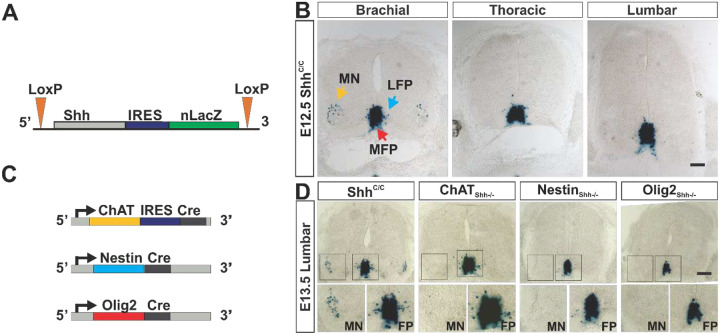
Shh expression and ablation strategy in the ventral spinal cord at E12.5. (**A**) Structure of the bicistronic and conditional nuclear (n)LacZ gene expression tracer allele for Shh. IRES: internal ribosome entry site; LoxP: recombination signals. (**B**) X-gal staining of Shh expressing cells of E12.5 control Shh^C/C^ spinal cord sections. Three sources of Shh identified: Medial floor plate (MFP, red arrow), lateral floor plate (LFP, blue arrow), and motor neurons (MN, yellow arrow). (**C**) Structure of the ChAT, Nestin, and Olig2 Cre driver alleles. Scale bar: 100μm. (**D**) X-gal staining revealing ablation of Shh at lumbar spinal cord levels at E13.5 in embryos with Shh ablation mediated by ChAT-Cre (ChAT_Shh_−/−), Nestin-Cre (Nestin_Shh_−/−) and Olig2-Cre (Olig2_Shh_−/−). Scale bar: 100μm.

Shh^C/C^ mice revealed a developmental stage- and spinal level- specific pattern of Shh expression in mice that was similar to the one observed in chick: At E12.5 Shh expression was evident at the ventral midline throughout the developing spinal cord and in prospective MNs of the lateral horns at brachial but not at thoracic or lumbar levels (Fig. 2B). From E13.5 onwards Shh is also expressed by lateral horn cells at lumbar levels (Fig. 2D). Shh expression is maintained postnatal in a subset of lateral horn cells at all levels (supplemental Fig. S1).

To investigate the specific role of Shh signaling from different cellular sources in the ventral spinal cord onto the pMN domain, we generated a series of mouse lines with conditional and in part overlapping patterns of Shh ablation. We used ChAT-Cre (ChAT_Shh_^−/−^) to target MNs, Nestin-Cre (Nestin_Shh_^−/−^) to target all VZD sources of Shh and Olig2-Cre (Olig2_Shh_^−/−^), to target MFP expression in addition to p3 and pMN domain derived Shh sources. We first analyzed the tissue specificity of Shh ablation by these Cre drivers. In ChAT_Shh_^−/−^ LacZ expression was strongly reduced in the lateral horns but had no apparent effect on floorplate expression (Fig. 2D) suggesting that the majority of Shh expressing lateral horn cells are nascent MNs. In contrast, and consistent with the previous reported expression of Nestin-Cre in all VZDs between E10.5 to E12.5 (Kramer et al., 2006), Nestin_Shh_^−/−^ mice showed a loss of LacZ expression in the ventral horns and in cells closely associated with the ventral midline (Fig. 2D). Further, consistent with Olig2 being expressed transiently in the MFP and P3 domain, and permanently in all pMN derived cells^27,44^, Olig2_Shh_^−/−^ exhibited reduced lacZ staining in the MFP, and absence of LacZ expression lateral to the midline (Fig. 2D).

**Fig. 3:**
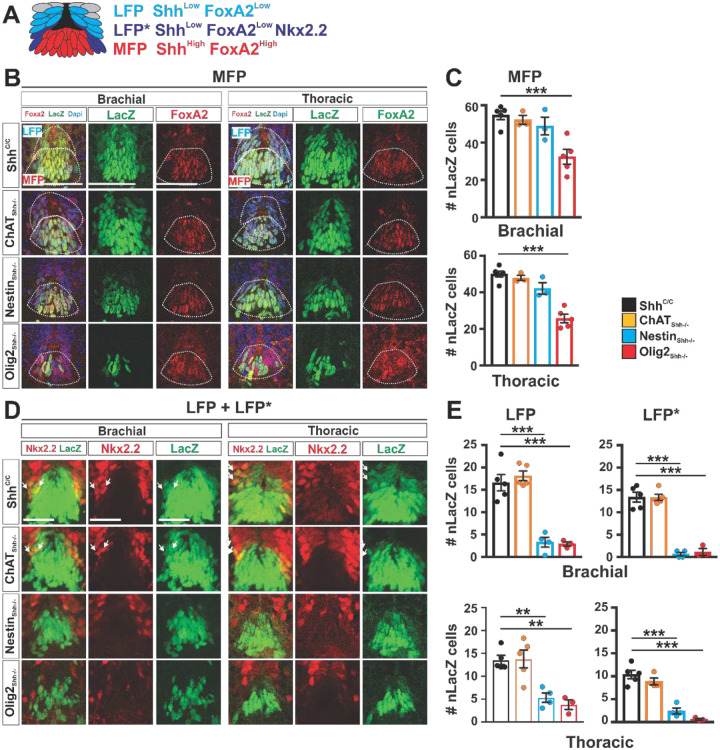
Shh expression and ablation in medial and lateral floorplate at E12.5. (**A**) Identification scheme used for quantifying Shh expression among medial (MFP) and lateral (LFP and LFP*) floorplate cells. (**B**) Immunostaining for LacZ and FoxA2 on brachial and thoracic segments. Identification of MFP (FoxA2+, LacZ+) and LFP (FoxA2+, LacZ+). LFP cells are identified as dorsal to MFP cells, oriented in tangent to MFP, and expressing lower levels of LacZ. Scale bar: 100 μm (**C**) Quantification of MFP LacZ recombination at brachial and thoracic segments for each Shh source per genotype. Brachial and thoracic segments Shh^C/C^ n=5, ChAT_Shh_^−/−^ n=3, Nestin_Shh_^−/−^ n=3, Olig2_Shh_^−/−^ n=5. Means ± SEM are shown. One-way ANOVA, Dunnett’s multiple comparison post hoc test. *p<0.05, **p<0.01, ***p<0.001. (**D**) Immunostaining for LacZ and Nkx2.2 on brachial and thoracic segments. Identification of LFP (Nkx2.2-, LacZ+) and LFP* (Nkx2.2+, LacZ+). Arrows point to migrating LFP* Nkx2.2+, LacZ+ cells. (**E**) Quantification of LFP and LFP* nLacZ recombination at brachial and thoracic segments for each Shh source per genotype. Brachial and thoracic segments Shh^C/C^ n=5, ChAT_Shh_^−/−^ n=4–5, Nestin_Shh_^−/−^ n=4, Olig2_Shh_^−/−^ n=3. Means ± SEM are shown. One-way ANOVA, Dunnett’s multiple comparison post hoc test. *p<0.05, **p<0.01, ***p<0.001.

**Fig. 4:**
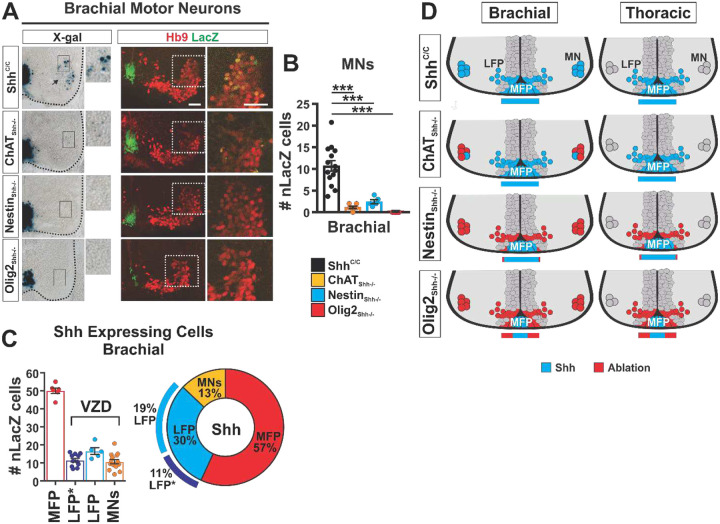
Shh expression and ablation in MNs at E12.5. (**A**) X-gal staining and immunostaining for LacZ and pan-MN marker Hb9 on brachial segments. Scale bars, 50 μm. (**B**) Quantification of LacZ recombination in brachial MNs. Shh^C/C^ n=14, ChAT_Shh_^−/−^ n=8, Nestin_Shh_^−/−^ n=5, Olig2_Shh_^−/−^ n=5. Means ± SEM are shown. One-way ANOVA, Dunnett’s multiple comparison post hoc test. *p<0.05, **p<0.01, ***p<0.001. (**C**) Numbers of LacZ expressing cells in MFP (FoxA2+) n=6, LFP (FoxA2+ Nkx2.2-) n=5, LFP* (Nkx2.2+) n=11, and among MNs (Hb9+) n=14. Ventricular zone derived (VDZ): LFP, LFP*, and MNs. Breakdown percentages of each Shh source relative to total. Means ± SEM are shown. (**D**) Schematic of Shh expressing cells at E12.5 brachial and thoracic segments and strategy of Cre ablation for each genotype.

Using detection of LacZ together with cell type specific marker expression we then quantified the numbers of Shh expressing cells of four distinct cell populations that together appeared to comprise most Shh expressing cells in the ventral spinal cord at E12.5: At brachial levels we found Shh in all cells of the MFP (defined by co-expression with FoxA2 and situated at the ventral midline stacking 4–5 cells high along the ventral to dorsal axis, Fig. 3A, B, C), LFP (defined by reduced levels of FoxA2 and Shh compared to MFP, situated immediately dorsal to the MFP and stacking about 3–4 cells high along the ventral to dorsal axis, Fig. 3A, D, E), LFP* (defined by co-expression of Nkx2.2 and Shh and situated in part at the lateral edges of the LFP or as isolated cells flanking the P3 domain, Fig. 3A, D, E), and motor neurons (MNs) of the lateral motor column (LMC, defined by co-expression with the pan MN marker Hb9 and lateral position in the ventral horns, Fig. 4).

In ChAT_Shh_^−/−^ embryos we found no indication of lacZ ablation in the MFP, LFP or LFP* at brachial or thoracic segments compared to controls (Fig. 3B–E). In Nestin_Shh_^−/−^ embryos LacZ expression was not affected in the MFP at brachial or thoracic levels (Fig. 3 B and C). However, in Nestin_Shh_^−/−^ embryos we found about 80% and 90% efficient ablation of LacZ among LFP and LFP* cells, resp. at brachial levels (Fig. 3 D and E). At thoracic levels of Nestin_Shh_^−/−^ embryos, Cre mediated recombination was less efficient and we found a 60 % reduction in LacZ prevalence among LFP cells and 77 % reduction among LFP* cells. (Fig. 3 D and E). In contrast to ChAT_Shh_^−/−^ and Nestin_Shh_^−/−^ embryos, in Olig2_Shh_^−/−^ embryos LacZ expression among MFP cells was reduced at brachial and thoracic levels (44 % and 39 % efficient recombination resp.) (Fig. 3 B and C). Further, in Olig2_Shh_^−/−^ embryos we found a 80 % and 90 % reduction in LacZ expression among LFP and LFP * cells, resp. at brachial levels and a 72% and 94% reduction in LacZ expression among LFP and LFP* cells, resp. at thoracic levels (Fig. 3D, E).

In ChAT_Shh_^−/−^ embryos we found a 80% reduction in the prevalence of LacZ expression among MNs at E12.5 (Fig. 4A). Expression of LacZ was also strongly attenuated in the remaining LacZ^+^ MNs compared to controls suggesting that locus recombination had also occurred in those cells but that residual LacZ was still present (Fig. 4A). In Nestin_Shh_−/− we found about 70 % efficient, and in Olig2_Shh_−/− near complete (98%) ablation of lacZ expression among MNs (Fig. 4B).

**Fig. 5:**
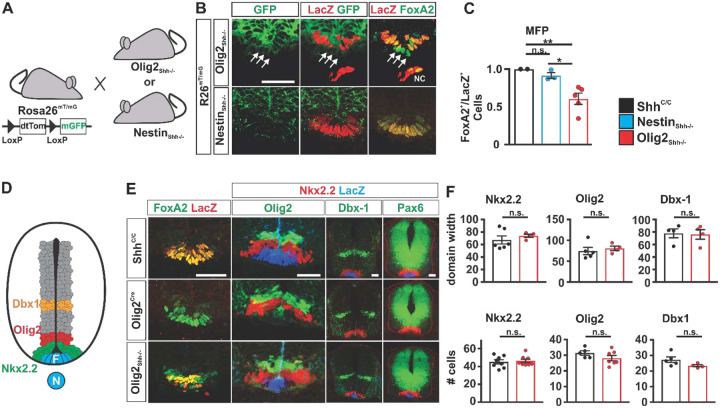
Early spinal cord patterning is unaffected in Olig2Shh−/−. (**A**) Detection of Cre activity in Olig2_Shh_^−/−^ and Nestin_Shh_^−/−^ mutants at E10.5 using the conditional R26mT/mG allele. (**B**) GFP co-labeling of Cre activity in addition to a loss of LacZ from Foxa2+ cells demonstrates Shh ablation in MFP of Olig2_Shh_^−/−^ but not Nestin_Shh_^−/−^ embryos. Arrows indicate MFP FoxA2+ cells that have lost nLacZ expression. NC, notochord. (**C**) Quantification of recombination frequency of the ratio of Foxa2+ nLacZ+ double positive cells. Shh^C/C^ n=2, Nestin_Shh_^−/−^ n=3, Olig2_Shh_^−/−^ n=5. Means ± SEM are shown. One-way ANOVA, Tukey post hoc test. *p<0.05, **p<0.01. (**D**) Scheme highlighting position of p3 (Nkx2.2), p0 (Dbx-1), and pMN (Olig2) domains relative to the MFP at E10.5. (**E**) Immunostaining of Shh-sensitive domains, Olig2, Nkx2.2, Dbx1, and Pax6 are unaffected at E10.5 in Olig2_Shh_^−/−^, despite MFP recombination. (**F**) Quantification of relative domain sizes and numbers of Nkx2.2, Olig2, Dbx1. Domain measurement Shh^C/C^ n=4–6, Olig2_Shh_^−/−^ n=4. Means ± SEM are shown. Cell counts Shh^C/C^ n=5–8, Olig2_Shh_^−/−^ n=4–8. Data were analyzed by Student’s t test. *p<0.05, **p<0.01.

Based on the numbers of LacZ expressing cells at E12.5 in the ventral neural tube at brachial levels in Shh^C/C^ controls we estimate that about 57% of all Shh producing cells are MFP cells, 19% are LFP cells, 11 % are LFP* cells and 13% are MNs (Fig. 4C). However, we noted that LacZ protein levels were distinctly lower in LFP and LFP* levels compared to the MFP and MNs (controls in (Fig. 5B, D and 4A), suggesting that the relative contribution of MNs to Shh production in the ventral spinal cord is larger than estimated by taking into account only cell numbers. The location of Shh expression in Shh^C/C^ controls and the varied degrees of ablation of Shh in ChAT_Shh_−/−, Nestin_Shh_−/− and Olig2_Shh_−/− embryos at E12.5 are schematically summarized in Fig. 4D.

We then investigated when Olig2-Cre and Nestin-Cre became active along the ventral midline. Using a conditional reporter allele (Fig. 5A), we found that Olig2-Cre is active in the MFP but not notochord at E10.5 resulting in the ablation of Shh from 46 ± 5.1%. of FoxA2+ cells (Fig. 5B and C). This ablation frequency was similar to the one observed at E12.5 in the MFP of Olig2_Shh_^−/−^ embryos (46 % at brachial and 39 % at thoracic levels, Fig. 3C) and therefore suggested that the degree of ablation of Shh from MFP in Olig2_Shh_^−/−^ animals is established at the time of the initiation of MN production and does not change significantly thereafter. Nestin-Cre activity let to a 8.5± 2.8% reduction in Shh expressing cells in the MFP that did not reach statistical significance.

The drastic loss of Shh expression in the MFP in Olig2_Shh_^−/−^ embryos at E10.5 prompted us to investigate a possible patterning defect along the ventral midline. However, consistent with previous reports that Shh expression by the notochord is sufficient for the establishment of precursor domains in the ventral spinal cord^25,29^ we found that the relative location and size of the p3- (Nkx2.2), pMN- (Olig2) and p0- (Dbx1) domains, and the location of the ventral border of the Pax6 expression domain are indistinguishable between Olig2_Shh_^−/−^ and control at E10.5 (Fig. 3D, E and F) and at E12.5 (Supplemental Fig. S2).

Together, these observations established a temporal order in which the three Cre drivers begin to effect Shh production in the ventral spinal cord with Olig2-Cre the earliest, followed by Nestin-Cre, followed by ChAT-Cre. Further, concordant with the onset and tissue specificity of expression, Olig2-Cre has the most drastic effect by ablating Shh from half of the MFP and from all VZDs, followed by Nestin-Cre, which ablates Shh from all VZDs but not MFP, followed by ChAT-Cre, which ablates Shh only from MNs.

### Ablation of Shh from MFP, but not VZD sources, impair MN generation.

**Fig. 6:**
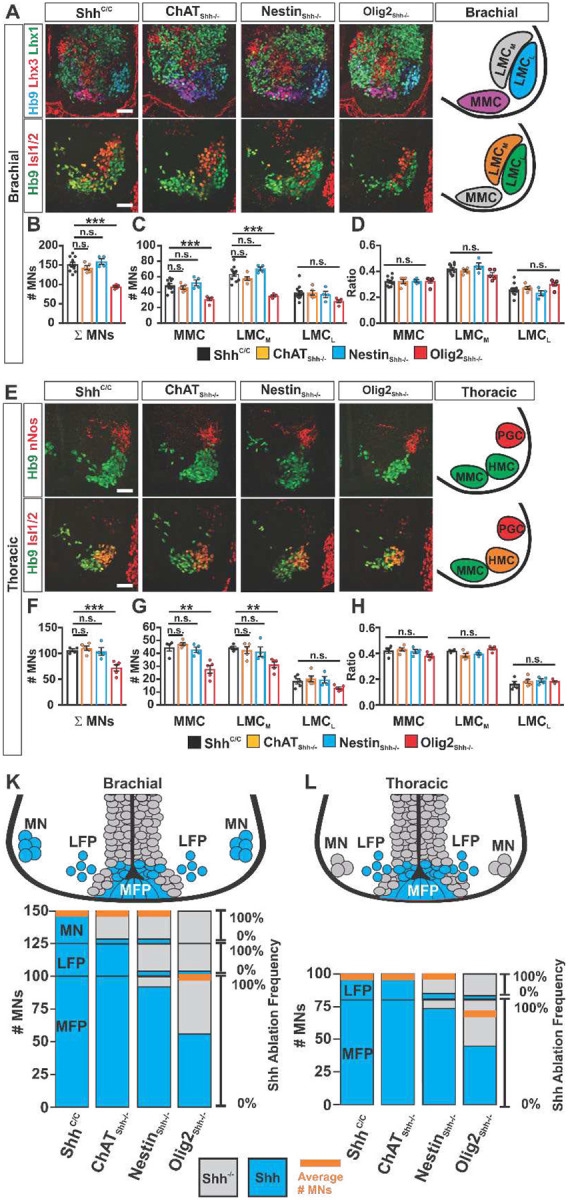
Shh signaling from MFP, but not VZD influences MN generation. (**A**) E12.5 brachial sections immunostained with Hb9, Lhx3, and Lhx1 to distinguish MMC and LMCL columns, and Hb9 and Isl1/2 to distinguish LMC_M_ and LMC_L_ columns. Scale bars, 50 μm. (**B**) Quantification of total brachial MNs. Shh^C/C^ n=11, ChAT_Shh_^−/−^ n=5, Nestin_Shh_^−/−^ n=4, Olig2_Shh_^−/−^ n=5. Means ± SEM are shown. One-way ANOVA, Dunnett’s multiple comparison post hoc test. *p<0.05, *p<0.01, ***p<0.001. (**C**) Quantification of total numbers of MMC, LMC_M_, and LMC_L_ MNs. Means ± SEM are shown. One-way ANOVA, Dunnett’s multiple comparison post hoc test. *p<0.05, **p<0.01, ***p<0.001. (**D**) Ratio of each motor column to total brachial MNs. Means ± SEM are shown. One-way ANOVA, Dunnett’s multiple comparison post hoc test. NS, not significant, P>0.5. (**E**) E12.5 thoracic sections immunostained with Hb9 and nNos to distinguish MMC and hypaxial motor column (HMC) from pre-ganglionic column (PGC), and Hb9 and Isl1/2 to distinguish MMC and HMC columns. Scale bars, 50 μm. (**F**) Quantification of total thoracic MNs. Shh^C/C^ n=4–5, ChAT_Shh_^−/−^ n=5, Nestin_Shh_^−/−^ n=4, Olig2_Shh_^−/−^ n=5. Means ± SEM are shown. One-way ANOVA, Dunnett’s multiple comparison post hoc test. *p<0.05, **p<0.01, ***p<0.001. (**G**) Quantification of total numbers of MMC, HMC, and PGC MNs. Means ± SEM are shown. One-way ANOVA, Dunnett’s multiple comparison post hoc test. *p<0.05, **p<0.01, ***p<0.001. (**H**) Ratio of each column to total thoracic MNs. Means ± SEM are shown. One-way ANOVA, Dunnett’s multiple comparison post hoc test. NS, not significant, P>0.5. (**K and L**) Top: schematic depiction of Shh expression (blue) at brachial and thoracic levels at E12.5. Bottom: Columns are composites of the average numbers of cells expressing Shh among MN, LFP and MFP cells stacked on top of each other. Schematic representing Shh ablation efficacy by each Cre driver and associated numbers of average MNs for brachial and thoracic sections.

We next investigated whether ablating Shh from VZD sources would impact the generation of MNs. We determined absolute numbers of MNs, and analyzed columnar pattern and relative distribution of MNs among columns at brachial and thoracic levels. We first visualized MN columnar organization by immunostainings for medial motor column (MMC) (Hb9+ Lhx3+), medial LMC (Hb9+ Isl1/2+), and lateral LMC (Hb9+, Isl1/2-, Lhx3-) at brachial levels (Fig. 6A) and MMC (Hb9+, Isl1−), hypaxial motor column (HMC) (Hb9+, Isl1/2+), and pre-ganglionic motor column (PGC) (Hb9-, nNos+) at thoracic levels (Fig. 6D) and found no apparent differences in the staining pattern either among ChAT_Shh_^−/−^, Nestin_Shh_^−/−^, or Olig2_Shh_^−/−^ compared to Shh^C/C^ controls. Consistent with unaffected MN positioning and columnar organization, we also find inconspicuous ventral root formation in Olig2_Shh_^−/−^ compared to Shh^C/C^ controls at E10.5 and E12.5. (Supplemental Fig. S3).

Quantification of MN numbers at E12.5 revealed no differences in ChAT_Shh_^−/−^ and Nestin_Shh_^−/−^ compared to Shh^C/C^ controls at brachial and thoracic levels. In contrast, in Olig2_Shh_^−/−^ we found a 38% reduction in the numbers of total MNs at brachial (Fig. 6B), and 32% at thoracic levels (Fig. 6F) compared to Shh^C/C^ controls. We then determined the relative numbers of MNs of the MMC (earliest born), medial LMC and lateral LMC (latest born). We found that MNs attained columnar identities in normal relative proportions in all genotypes (Fig. 6C and F)

The results of these experiments are summarized in Fig. 6G where we associate the reduction in the numbers of MNs (red line in each column) with the tissue specific efficiency of Shh ablation (greyed out area of the otherwise blue columns) in MNs, LFP and MFP in ChAT_Shh_^−/−^, Nestin_Shh_^−/−^, and Olig2_Shh_^−/−^ at brachial and thoracic levels, which was quantified in Fig. 3 and 4). The 80% efficient ablation of Shh from MNs in ChAT_Shh_^−/−^ as well as the 80 – 90% efficient ablation of Shh from MNs and LFP, resp. in Nestin_Shh_^−/−^, had no effect on MN numbers at brachial or thoracic levels at E12.5. In contrast, the 70 % and 98 % efficient ablation of Shh from LFP and MNs resp. together with a 44% efficient ablation of Shh from MFP in Olig2_Shh_^−/−^ results in a 30% reduction of MN numbers at brachial and thoracic levels (Fig. 6 G, **orange line**). Together these results revealed that MN production and columnar diversification becomes impacted by partial reductions in Shh signaling from the MFP but can proceed in the absence of Shh signaling from MNs and LFP. Thus, these results suggested that ChAT_Shh_^−/−^ and Nestin_Shh_^−/−^ embryos are informative paradigms to test the function of MN produced Shh on oligodendrogenesis.

### Shh from MNs becomes progressively more critical for maintaining a larger pMN domain at brachial levels prior to oligodendrogenesis.

**Fig. 7:**
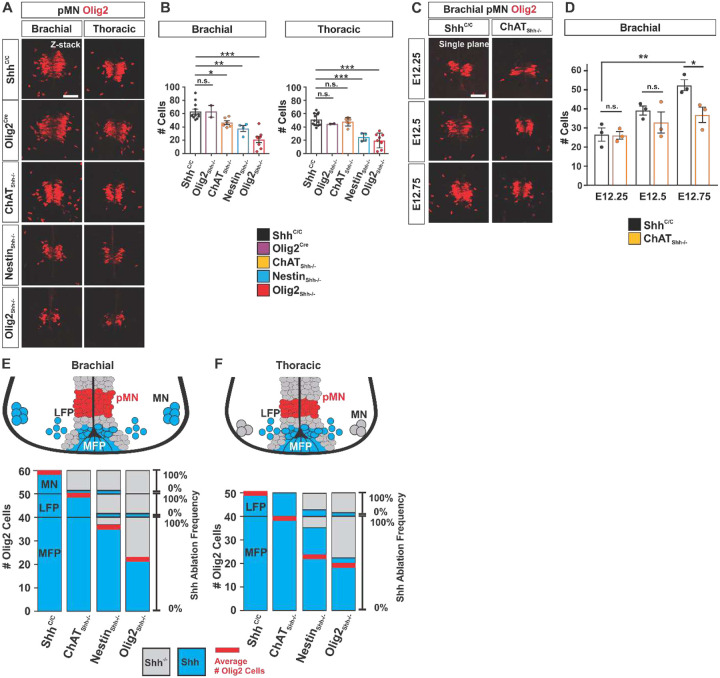
Shh from VZD in addition to MFP is critical for pMN domain maintenance in a spinal level specific manner. (**A**) Immunostaining for Olig2 in the pMN domain along the AP axis at brachial and thoracic levels in Shh^C/C^, Olig2-Cre, ChAT_Shh_^−/−^, Nestin_Shh_^−/−^, and Olig2_Shh_^−/−^ embryos. Scale bars, 50 μm. (**B**) Quantification of numbers of Olig2 cells in the pMN domain at brachial and thoracic sections. Means ± SEM are shown. Shh^C/C^ n=12, Olig2-Cre n=2, ChAT_Shh_^−/−^ n=6, Nestin_Shh_^−/−^ n=4, Olig2_Shh_^−/−^ n=8. One-way ANOVA, Dunnett’s or Tukey’s multiple comparison post hoc test. *p<0.05, **p<0.01, ***p<0.001. (**C**) Expansion of pMN domain at onset of gliogenesis at brachial segments between E12.25-E12.75 is reduced in ChAT_Shh_^−/−^. Scale bars, 50 μm. (**D**) Quantification of Olig2 cells in the pMN domain on brachial segments. Means ± SEM are shown. E12.25 Shh^C/C^ n=3, ChAT_Shh_^−/−^ n=3, E12.50 Shh^C/C^ n=3, ChAT_Shh_^−/−^ n=3, E12.75 Shh^C/C^ n=3, ChAT_Shh_^−/−^ n=3. Data were analyzed by Student’s t test. *p<0.05. (**E and F**) Top: schematic depiction of Shh expression and relative size and location of pMN domain at brachial and thoracic levels at E12.5. Bottom: Schematic representing Shh ablation within the genotypes and associated numbers of average pMN Olig2 cells at brachial and thoracic levels. Blue columns indicate percent of Shh expressing cells normalized to Shh^C/C^ controls for MFP, LFP, and MNs. Grey areas indicate Cre ablation efficacy. Red bars indicate average numbers of Olig2 cells within the pMN.

We next quantified the effect of Shh ablation on the numbers of Olig2^+^ cells present at E12.5 in the pMN domain (pMN_Olig2_^+^) in ChAT_Shh_^−/−^, Nestin_Shh_^−/−^, and Olig2_Shh_^−/−^ mutant embryos compared to Shh^C/C^ and Olig2-Cre controls. Consistent with Shh expression by brachial but not by thoracic MNs, we found a 26% decrease in the numbers of pMN_Olig2_^+^ cells specifically at brachial but not at thoracic levels in ChAT_Shh_^−/−^ embryos (Fig. 7A and B). Since we observed an increase in Shh expression during LMC MN maturation (Fig. 1), we quantified the effect of MN_Shh_ on the numbers of pMN_Olig2_ cells with greater temporal resolution. Consistent with the possibility that Shh signaling emanating from LMC MNs becomes progressively more impactful on the pMN domain over time, we found a progressively greater reduction in the numbers of pMN_Olig2_ cells from E12.25 to E12.75 (Fig. 7C and D). This period is marked by the rapid enlargement of the pMN domain in controls (Fig. 7C and D), suggesting that MN_Shh_ might become increasingly critical as the pMN domain expands towards the end of neurogenesis. In contrast to ChAT-Cre, the ablation of Shh by Nestin-Cre reduces the numbers of pMN_Olig2_^+^ cells at both, brachial and thoracic levels (40% and 51%, resp.) (Fig. 7A and 7B). Olig2-Cre driven ablation of Shh causes a reduction in the numbers of pMN_Olig2_^+^ cells at all spinal levels as well, but the effect is more severe compared to Nestin-Cre driven Shh ablation with a decrease in pMN_Olig2_^+^ cells of 66% at brachial, and 61% at thoracic levels (Fig. 7A and B).

The results of these experiments are summarized in Fig. 7E where we associate the numbers of pMN_Olig2_^+^ cells at brachial and thoracic levels (red line in each column) with the specific efficiency of Shh ablation in ChAT_Shh_^−/−^, Nestin_Shh_^−/−^ and Olig2_Shh_^−/−^ embryos (greyed out area in each otherwise blue column), which was quantified in Fig. 3 and 4. We found 20% more pMN_Olig2_^+^ cells at brachial than thoracic segments in controls (Fig. 7E, Shh^C/C^ columns). Importantly, the 80% efficient ablation of Shh from MNs at brachial levels in ChAT_Shh_^−/−^ (Fig. 7E, greyed out area in column ChAT_Shh_^−/−^), reduces the numbers of pMN_Olig2_^+^ cells at brachial levels to those present in controls at thoracic levels (Fig. 7E, red lines). At brachial levels, Nestin_Shh_^−/−^ embryos exhibited near complete ablation of Shh from MNs and LFP (Fig. 7E, greyed out area in column Nestin_Shh_^−/−^) and reduced the numbers of pMN_Olig2_^+^ cells to 40 % (Fig. 7E, red lines), suggesting that MN_Shh_ and LFP_Shh_ have similar and additive effects on the size of the pMN_Olig2_^+^ cell population. Further, ablation of Shh from all VZD and from about half of the MFP in Olig2_Shh_^−/−^ embryos (Fig. 7E, greyed out area in column Olig2_Shh_^−/−^) resulted in 66% reduction in the numbers of pMN_Olig2_^+^ cells (Fig. 7E, red line). At thoracic levels, we found a similar magnitude in the reduction of the numbers of pMN_Olig2_^+^ cells in Nestin_Shh_^−/−^ and Olig2_Shh_^−/−^ (Fig. 7E, red line) despite of a much greater total reduction in numbers of Shh producing cells by Olig2-Cre compared to Nestin-Cre (Fig. 7E, greyed out area in otherwise blue column), suggesting a greater significance of LFP_Shh_ for the size of the pMN_Olig2_^+^ population at thoracic levels compared to brachial levels.

### Precocious exhaustion of the pMN_Olig2_^+^ cell population by reduced Shh signaling results in unrecoverable diminishment of OPC production.

**Fig. 8:**
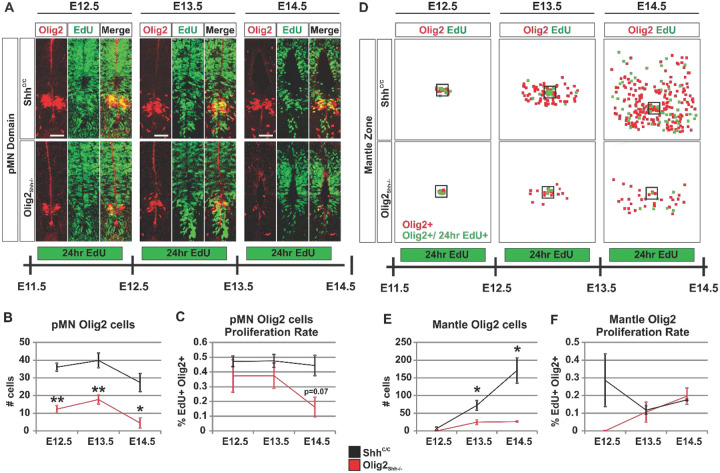
Exhaustion of pMN_Olig2+_ cells results in diminished OPC production. (**A**) pMN domain proliferation in lumbar sections labeled by EdU incorporation in 24hr intervals for E12.5- E14.5 in Shh^C/C^ and Olig2_Shh_^−/−^. Scale bar: 50 μm. (**B**) Total Olig2 cells in pMN at E12.5-E14.5. Means ± SEM are shown. Shh^C/C^ n=3–4, Olig2_Shh_^−/−^ n=3–4. Data were analyzed by Student’s t test. *p<0.05, **p<0.01. (**C**) Proliferation rate of Olig2 cells in pMN E12.5, E13.5, and E14.5. Means ± SEM are shown. Shh^C/C^ n=3–4, Olig2_Shh_^−/−^ n=3–4. Data were analyzed by Student’s t test. (**D**) Tracing of migrating Olig2 cells on representative lumbar sections with proliferation labeled by EdU incorporation in 24hr intervals between E12.5- E14.5. Box indicates pMN domain, Olig2+ cells in the mantle zone (red), Olig2+ EdU+ cells (green). (**E**) Total Olig2 cells in mantle zone at E12.5, E13.5, and E14.5. Olig2 cell numbers in Olig2_Shh_^−/−^ mantle remain reduced. Means ± SEM are shown. Shh^C/C^ n=3–4, Olig2_Shh_^−/−^ n=3–4. Data were analyzed by Student’s t test. *p<0.05. (**F**) Proliferation rate of Olig2 cells in mantle zone at E12.5, E13.5, and E14.5. Olig2 cells that have migrated out of the pMN in Olig2_Shh_^−/−^ mutants proliferate at the same rate as controls. Means ± SEM are shown. Shh^C/C^ n=3–4, Olig2_Shh_^−/−^ n=3–4.

The diminished population of pMN_Olig2_^+^ cells in Shh mutants could recover during OPC production by increased precursor recruitment from dorsal ventricular precursor domains^57^, proliferation of remaining pMN_Olig2_^+^ precursor cells, or increased differentiation and amplification of OPC fated cells that have left the pMN domain. We therefore quantified the size of the pMN_Olig2_^+^ population at the end of ventral oligodendrogenesis at E14.5. We found an almost complete absence of pMN_Olig2_^+^ cells in Olig2_Shh_^−/−^ compared to Shh^C/C^ controls suggesting that increased recruitment of precursors to the pMN domain did not occur in mutants (Fig. 8A and B). We then determined whether pMN_Olig2_^+^ cells and/or migrating OPCs in Olig2_Shh_^−/−^ increase their rate of proliferation during the phase of OPC production compared to controls. We injected EdU into pregnant dams at E11.5, E12.5, and E13.5 and quantified the numbers of pMN_Olig2_^+^ cells 24h later. We found comparable and broad EdU+ labeling throughout the ventricular zone of Olig2_Shh_^−/−^ mutants and controls, suggesting overall progenitor proliferation was not affected in mutants (Fig. 8A). We then compared the dynamics of pMN_Olig2_^+^ cell maintenance in Shh^C/C^ controls and Olig2_Shh_^−/−^ mutants. In controls, we observed a ~25% decrease in the numbers of pMN_Olig2_^+^ cells over the course of OPC production from E12.5 to E14.5 (Fig. 8B). In contrast, in Olig2_Shh_^−/−^ mutants numbers of pMN_Olig2_^+^ cells declined to near undetectable levels during the same period indicating a precocious exhaustion of the pMN_Olig2_^+^ cell population during OPC generation (Fig. 8B). We next determined the number of proliferating pMN_Olig2_^+^ cells in controls and mutants. In controls, we found that 47% of pMN_Olig2_^+^ cells incorporated EdU at E12.5, E13.5 and E14.5. In contrast, in Olig2_Shh_^−/−^ embryos the number of proliferating pMN_Olig2_^+^ cells was reduced to 37% at E12.5 and E13.5, followed by a further decrease to 16% by E14.5 (Fig. 8C). These results indicate that the exhaustion of the pMN_Olig2_^+^ cell population is associated with a reduced proliferation of precursor cells during OPC production, which is compounded by reduced numbers of pMN_Olig2_^+^ cells that are present at the beginning of OPC production.

We next tested whether OPC production in Olig2_Shh_^−/−^ mutants recovers through increased amplification of precursors once they have exited the pMN domain. We analyzed numbers of EdU^+^ Olig2^+^ cells in the mantle zone (Fig. 8D). While there were very few Olig2^+^ cells in the mantle zone of mutants and controls at E12.5, with ongoing production and expansion of these cells, we found a 3-fold and 15-fold reduction of Olig2^+^ cells at E13.5 and E14.5, resp., in mutants compared to controls (Fig. 8E). The proportion of OPCs in the mantle zone that incorporated EdU in Olig2_Shh_^−/−^ was similar at E13.5 and E14.5 compared to controls suggesting that OPCs in mutants do not amplify at an increased rate compared to controls (Fig. 8F). The cells that did emerge from the pMN in Olig2_Shh_^−/−^ disperse as rapidly as their control counterparts, resulting in a ventral spinal cord that is populated with nascent OPCs with a 15-fold lower density compared to controls (Fig. S4). Additionally, we examined OPC number and EdU incorporation at E14.5 in thoracic and lumbar segments in the mantle- and white matter- areas. We found significantly reduced numbers of OPCs at lumbar levels and no indication for increased amplification of the remaining OPCs (Fig. S5). These results revealed that the yield of the pMN domain during OPC production is significantly determined by the numbers of OPCs that settle in white and grey matter.

## Discussion

**Fig. 9:**
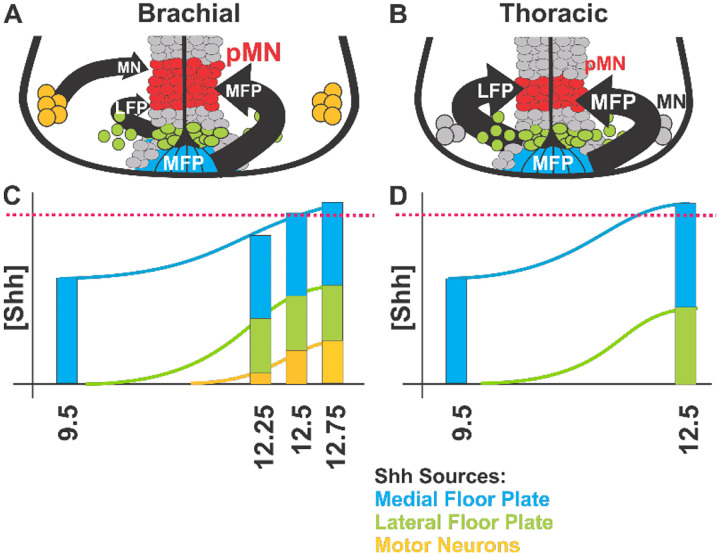
Initiation of oligodendrogenesis in the ventral spinal cord requires multiple and spinal level specific sources of Shh. (**A and B**) Schematic representation of location and actions of Shh sources at brachial and thoracic levels. (**C and D**) Relative contribution of ventral sources of Shh signaling at brachial and thoracic levels. Red dotted line: threshold of Shh signaling strength triggering oligodendrogenesis.

During early neural tube development, Shh emerges sequentially from the ventral midline structures notochord, MFP and LFP resulting in the spatio-temporal patterning and subsequent maintenance of five ventral precursor domains along the entire spinal neuraxis^21^. Here, we investigated the contributions of Shh expressed by nascent MNs of the lateral motor column (LMC) to ventral neural tube development. We provide evidence that LMC derived Shh is critical for forming and maintaining a larger and more yielding Olig2^+^ precursor cell population in the pMN domain during OPC production at brachial compared to thoracic levels (Fig. 9). Our data suggests that the dependence of OPC production on Shh expression by LMC MNs results in the scaling of pMN domain output along the rostro-caudal axis during OPC production and might provide a safety mechanism that ensures OPC generation commences after LMC completion.

One of our main findings is that selective ablation of Shh expression from MNs reduces the size of the pMN_Olig2+_ precursor cell population present at brachial levels to the smaller size present at thoracic levels at the beginning of oligodendrogenesis. Why can panMN ablation of Shh achieved by ChAT-Cre result in a spinal level restricted phenotype? Key here is that Shh expression by MNs at the transition from neurogenesis to oligodendrogenesis is restricted to the LMC, which forms at limb levels only^15,58–61^, while all other sources of Shh in the developing neural tube are present at all segmental levels. Our observations reveal that the induction of MN_Shh_ (1) is embedded in post mitotic MN maturation, (2) occurs towards the end of MN generation and prior to oligodendrogenesis, and (3) results in a pattern of Shh signaling that breaks the otherwise monotonous expression of Shh by MFP and LFP along the rostro-caudal axis.

Isolating the function of MN_Shh_ at the beginning of oligodendrogenesis required the efficient and selective ablation of Shh from MNs without effecting earlier developmental processes. Taking advantage of a bi-cistronic LacZ tracer for Shh expression in mouse embryos we reveal no evidence for Shh ablation by ChAT-Cre from other sources than MNs in the ventral neural tube (Fig. 4). Importantly, our analysis of ChAT_Shh_−/− embryos revealed that MN_Shh_ does not play a role in MN generation and columnar specification (Fig. 6). This result is concordant with the dynamics of Shh expression which becomes only maximal at the end of motor neuron genesis and lags behind the expression of MN columnar markers (Fig. 1). Further, the progressive increase in MN_Shh_ expression in the ventral horns towards the end of MN production is mirrored by the progressive impoverishment of the Olig2+ precursor cell population in the pMN domain of ChAT_Shh−/−_ embryos from E12.25 to E12.75 (Fig. 7E) suggesting that MN_Shh_ becomes progressively more critical as the developing spinal cord rapidly enlarges during this period. The finding that Ptc1 expression in the lateral horns is much weaker than expected by the strong expression of Shh by MNs (Fig. 1) supports a direct mechanism by which MN_Shh_ impinges on the pMN domain. Consistent with this possibility it has been found previously that nascent neurons that emigrate from the ventricular zone remain in temporary contact with the neuroepithelium via trailing microtubule-based cilia and/or cytonemes from which Shh could be secreted onto pMN precursor cells^62,63^. It will be of interest to determine by which means MN_Shh_ becomes available to pMN cells in future experiments.

It was previously found that the onset of OPC production is determined by an increase of Shh signaling strength in the pMN domain^32,33^. Expanding on this result, our findings reveal that the mechanisms by which oligodendrogenesis triggering increases in Shh are generated differ between brachial and thoracic levels (Fig. 9). Specifically, at brachial but not at thoracic levels Shh signaling increases dynamically as a function of the progressive production of MNs of the LMC which turn on Shh expression once they have migrated into the ventral horns. Linking previous MN production and maturation to subsequent oligodendrogenesis provides a quality control mechanism that could ensure that oligodendrogenesis can only start upon completion of LMC formation. Consistent with our finding that MN_Shh_ is specifically needed for adapting OPC production to populating the spinal cord selectively at limb levels, it was found that MNs are not necessary for the production of OPCs in Zebrafish^64^.

To further gauge the significance of MN_Shh_ to pMN activity, we compared the effect of MN_Shh_ to all Shh that can be produced by ventricular zone derivatives (VZD_Shh_, i.e. MN and LFP) using Nestin-Cre mediated ablation of Shh. Monitoring LacZ expression upon Nestin-Cre ablation revealed that more than one third of all Shh expressing cells are ventricular zone descendants in mouse embryos at E12.5 (Fig. 4). Ablation of VZD_Shh_ has no effect on MN generation and columnar specification (Fig. 6). However, LFP_Shh_ is critical for the maintenance of the pMN_Olig2+_ precursor cell population at brachial and trunk levels consistent with Shh expression in the LFP at all spinal levels and previous work^21,22,32,35^. Interestingly, the relative contribution of LFP_Shh_ to the regulation of the size of the pMN_Olig2+_ precursor cell population is greater at thoracic levels than brachial levels (Fig. 7 and Fig. 9). These observations suggest that MN_Shh_ does not only add to the total Shh that impinges onto the pMN domain but might have additional, selective functions. Consistent with that speculation, we found that the dorsal most aspects of the pMN domain is particular sensitive to reduction in Shh and is almost completely devoid of Olig2+ expressing cells at the end of MN production at E12.5 in Nestin_Shh_^−/−^ and Olig2_Shh_^−/−^ embryos (supplemental Fig. S6). This area of the pMN domain is not only the furthest dorsal from midline sources of Shh, but must also be the entry zone where ventrally migrating precursor cells enter the pMN domain at the beginning of oligodendrogenesis^57^. It is thus possible that MN_Shh_ is selectively needed to allow OPC forming precursor cells to enter the pMN domain at brachial levels. Cellular heterogeneity among pMN_olig2_+ cells was recently found by single cell profiling in Zebrafish^64,65^. In future experiments, it might be of interest to determine whether cell heterogeneity in brachial and thoracic pMN domains in mice is differentially affected by ablation of Shh from MNs and LFP.

Our current study does not rule out that MN_Shh_ also plays a role in establishing spinal level appropriate OPC density and function at later stages after OPC production. Indeed, it was recently found that OPCs proliferate without differentiating into myelinating oligodendrocytes when forced to express a constitutive active form of the obligate necessary Shh signaling effector smoothened (SmoM2)^66^. Thus, MN_Shh_ might have at least two functions during embryonic development: early Shh expression by LMC MNs is needed for forming a larger pMN domain at limb levels during OPC production and later MN_Shh_ might contribute to the mitotic expansion of the OPC population. Both functions seem critical since Olig2_Shh−/−_ mice die at 3 weeks of age and harbor OPCs in the ventral spinal cord that appear unable to communicate appropriately with injured MNs^43^. Our study highlights the need to elucidate spinal level specific OPC generation in greater molecular detail in order to guide systems and disease specific in vitro production of OPCs for replacement therapies.

## METHODS

The procurement information for all reagents and mouse strains used in this study is listed in Table 1 in supplemental information.

### Transgenic mice.

All animal experiments were approved by the Institutional Animal Use Care Committee at CUNY. The following mouse strains were used and genotyped as described previously: Shh-nLZ^L/+^ animals (Gonzalez-Reyes et al., 2012), Chat-Cre (Rossi et al., 2011), Olig2-Cre (Dessaud 2007), Nestin-Cre (Tronche et al., 1999), Rosa26^mT/mG^ (Muzumdar et al., 2007). Mice were maintained on a C57BL/6 background. Noon on the day of the plug was considered E0.5. Mice were kept on a 12 hr dark/light cycle and the day of birth designated P1. For E12.25, E12.50, and E12.75 pMN analysis, pregnant dams were sacrificed at E12.5 according to plug date and embryos were binned into three groups (E12.25, E12.50, and E12.75), based on how many Olig2+ cells have migrated out of the pMN domain. Embryo sections which had an average of less than 5 cells migrate out of the pMN were considered 12.25, 5–20 cells migrating out – E12.50, and >20 cells migrating out - E12.75.

### In vivo EdU assay.

Pregnant dams received EdU (5-ethynyl-20 -deoxyuridine, Invitrogen) dissolved in PBS by intraperitoneal injection (50 mg/kg) and sacrificed after 24 hours. Tissue sections were stained using the Click-iT Plus EdU Alexa Fluor 647 Imaging Kit (Thermofisher).

### Tissue Processing.

All mice were sacrificed using an overdose of anesthetic, subjected to transcardial perfusion with 4% (w/v) paraformaldehyde (PFA) in 0.1 M PBS pH 7.4. Spinal cords and embryos were dissected, postfixed in 4% PFA for 1 hr at 4°C, cryoprotected with 30% (w/v) sucrose in 0.1M PBS for 24–48 hr, embedded and frozen in OCT medium, and stored at −80°C. Tissues were sectioned at 20 μm and collected onto glass slides.

### Immunocytochemistry and microscopy.

20um thick spinal cord cryosections were air dried for 30 min. Then sections were washed with PBS for 10 mins and with 0.3% [v/v] Triton X-100 in PBS for 20 min. Sections were then pre-treated with blocking solution (10% [v/v] horse serum and 0.3% [v/v] Triton X-100 in PBS) for 90 mins and incubated with primary antibodies overnight at 4C. The next day, following 3 PBS washes the sections were incubated with secondary antibodies for 2 hr at room temperature. A list of all antibodies and compounds used is provided in a table. For cell counts, at least three sections per animal from at least three mice were examined, unless otherwise noted. Images were acquired using a Zeiss LSM880 confocal microscope.

### Experimental design and statistical analysis.

For each analysis, results form independent embryos were treated as biological replicates and analyzed using Prism 7 (Graphpad Software Inc.). Analysis of multiple groups was made using one-way ANOVA followed by the Tukey or Dunnett’s post hoc analysis tests. For 2-groups analyses, unpaired Student’s t test was used. The data are presented graphically as: *(p < 0.05), **(p < 0.01), and ***(p < 0.001).

## Supplementary Material

1

## Data Availability

All data associated with this study are available from the corresponding author upon request.

## Main Points:

- Sonic Hedgehog (Shh) becomes expressed by motor neurons of the Lateral, but not Medial, Motor Column at the end of motor neuron production. Motor neuron specific expression of Shh at limb levels breaks the otherwise monotonous production of Shh along the rostro-caudal axis.
- Motor neuron expressed Shh is critical for producing and maintaining a larger and more yielding pMN precursor domain during oligodendrogenesis at limb levels compared to trunk levels.
- Reduced oligodendrocyte precursor production due to diminished Shh signaling is not rescued by increased proliferation of remaining OPCs.
