# Supplementary material for: Lateral Motor Column specific expression of Sonic Hedgehog contributes to maintenance and scaling of pMN progenitor cell populations during oligodendrogenesis
Source: Res Sq. 2024 May 13:rs.3.rs-4249282. Preprint. [Version 1] doi: 10.21203/rs.3.rs-4249282/v1 (PMC11118686; doi:10.21203/rs.3.rs-4249282/v1)
Supplement: 1 [file NIHPPRS4249282V1-supplement-1.pdf]

Supplementary Information for

"Lateral Motor Column specific expression of Sonic Hedgehog contributes to maintenance and scaling of pMN progenitor cell populations during oligodendrogenesis"

Lev Starikov, Miruna Ghinia-Tegla, and Andreas H. Kottmann

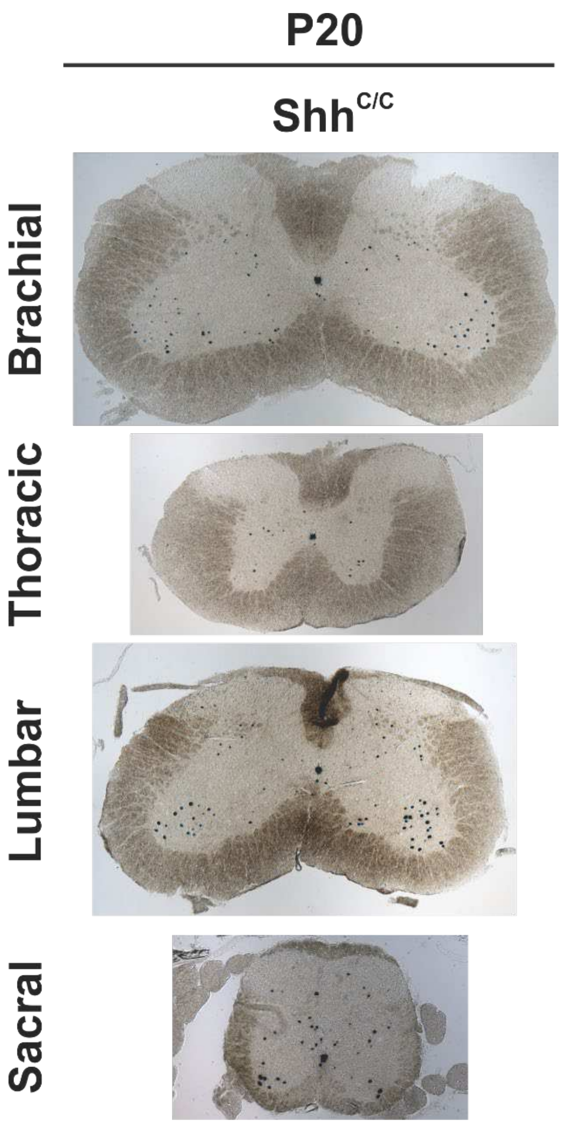

Supplementary Figure 1: Shh expression in MNs.

(**A**) X-gal staining revealing Shh expression pattern throughout AP axis of P20 control $Shh^{C/C}$ spinal cords at brachial, thoracic, lumbar, and sacral segments.

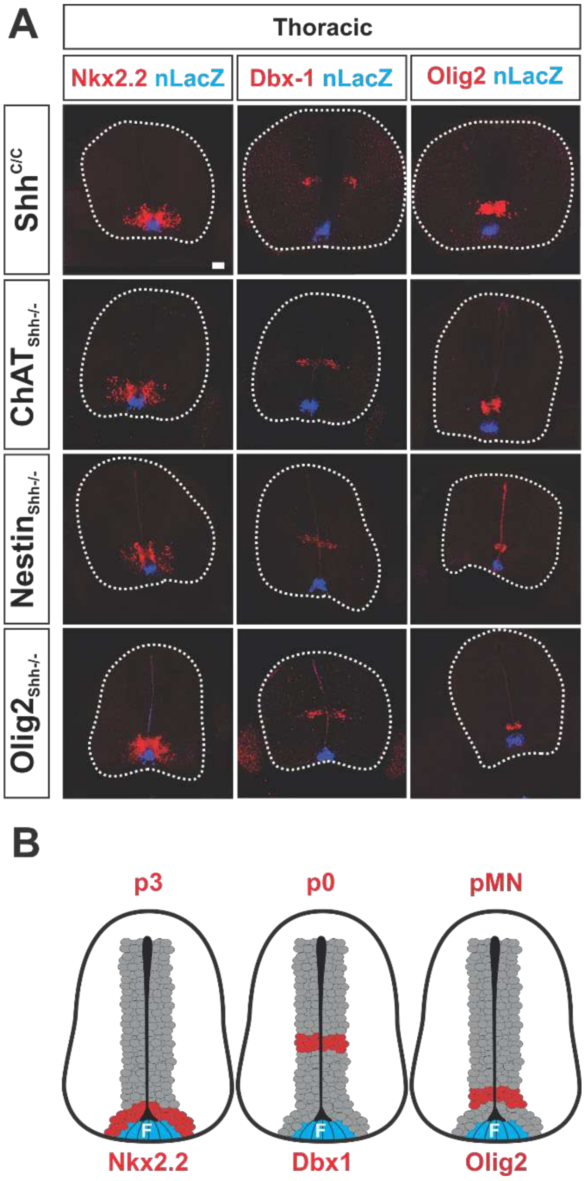

Supplementary Figure 2: Ventral precursor domains are unaffected at E12.5:

(A) Immunostaining of nLacZ with Nkx2.2, Dbx-1, and Olig2 on E12.5 thoracic sections. Scale bars: 50 µm.
(B) Schematic depiction of the p3, p0 and pMN domain in the thoracic neural tube at E12.5.

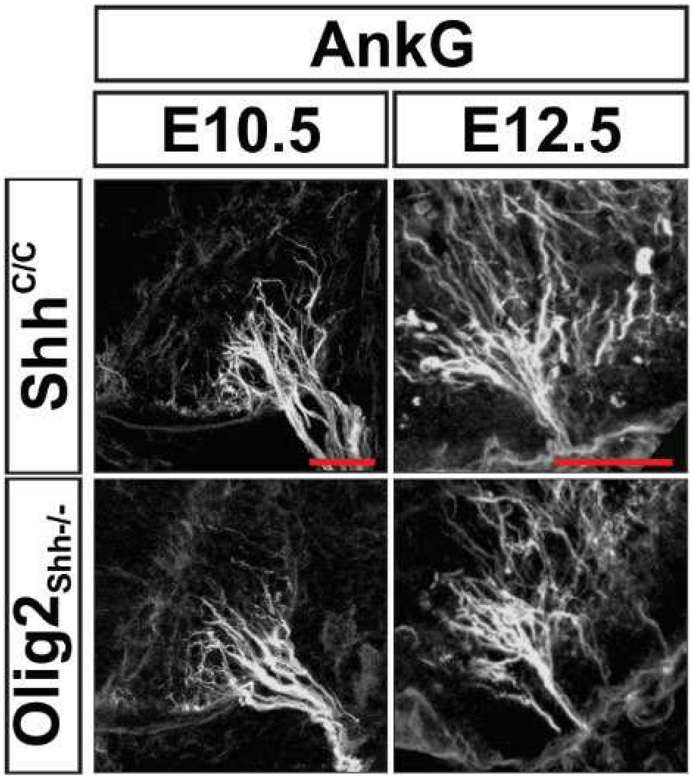

Supplementary Figure 3: AnkG staining at E10.5 and E12.5 revealing correct MN axon fasciculation and exit from ventral horns in $Olig2_{Shh}^{-/-}$. Scale bars: 25 μm.

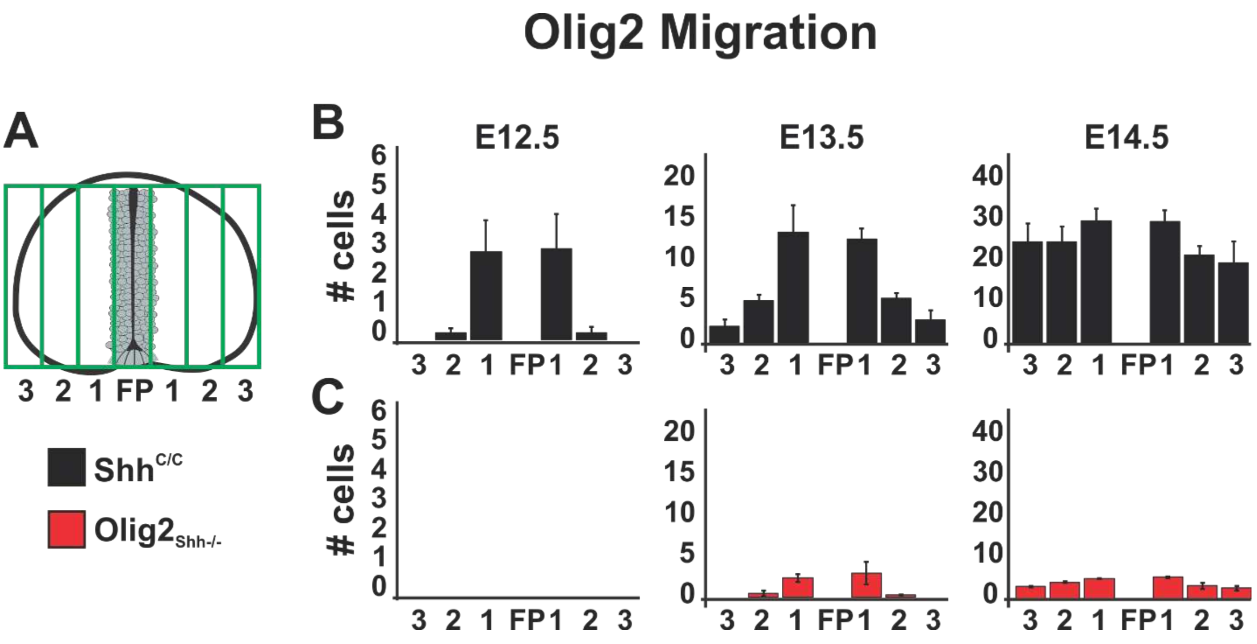

Supplementary Figure 4: Analysis of migrating Olig2 cell dispersion in Olig2$_{Shh}^{-/-}$ mutant and control embryos.

Lumbar spinal cord sections from E12.5-E14.5 were binned into 6 zones excluding the ventricular zone, and numbers of Olig2 cells in each zone were quantified. Means ± SEM are shown. Shh$^{C/C}$ (n = 3-4 embryos), Olig2$_{Shh}^{-/-}$ (n = 3 embryos).

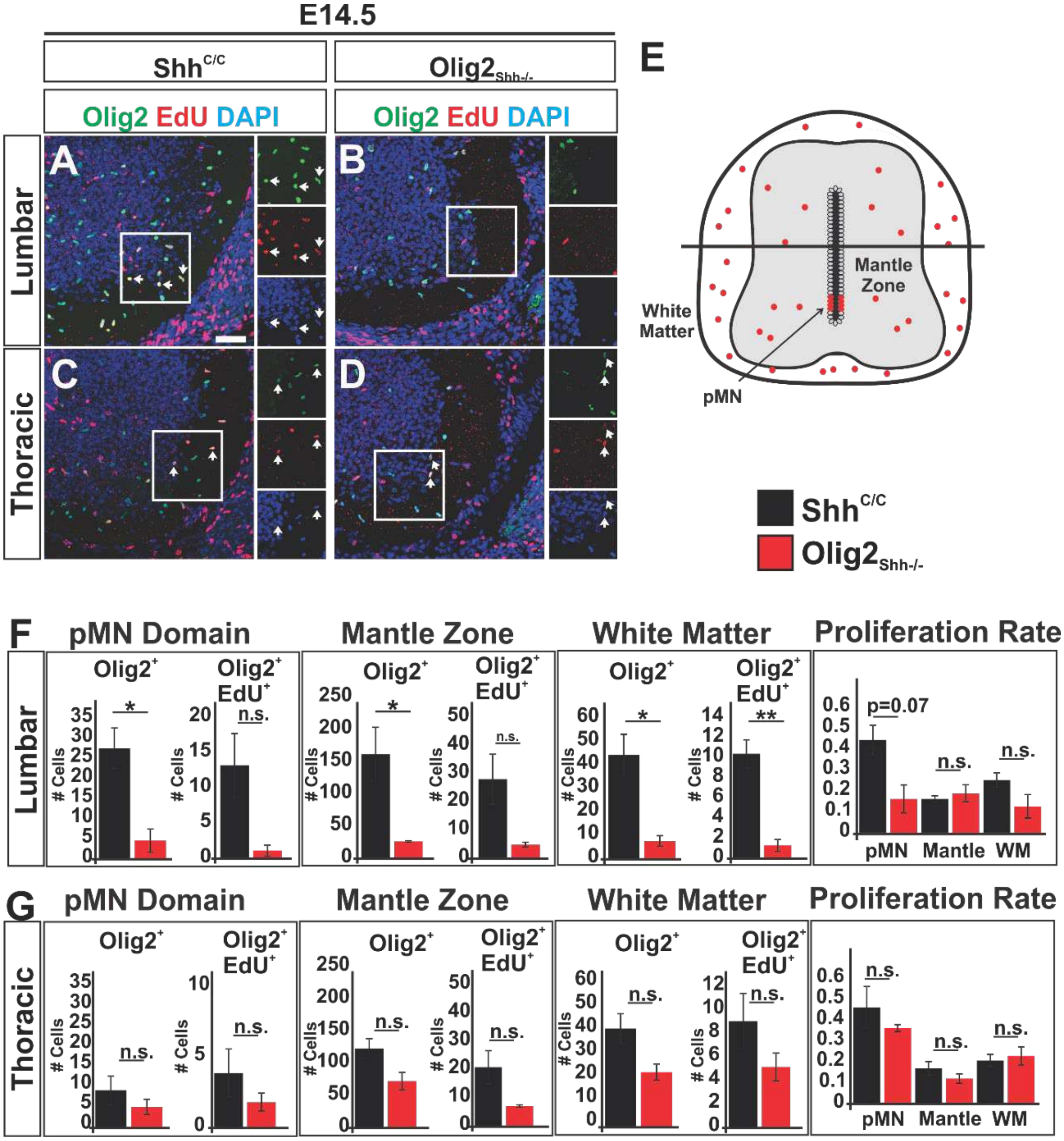

Supplementary Figure 5: OPCs are reduced and do not increase proliferation rate.

(**A-D)** Immunostaining on E14.5 sections for Olig2, EdU, and DAPI at (**A and B**) lumbar and (**C and D**) posterior thoracic. Arrows indicate co-expression of Olig2 and EdU.
(**E**) Schematic depicting areas analyzed.
(**F and G**) 24hr pulse chase with a single EdU injection to label proliferating Olig2 cells. Total Olig2 cells and Olig2+ Edu+ double positive cells are reduced in the pMN, mantle zone, and white matter at both (**F**) lumbar and (**G**) thoracic segments in $Olig2_{Shh}^{-/-}$ compared to control. $Shh^{C/C}$ (n = 3 embryos), $Olig2_{Shh}^{-/-}$ (n = 3 embryos). Means ± SEM are shown. Data were analyzed by Student's t test. $*p < 0.05$, $**p<0.01$. Scale bar, 50 µm.

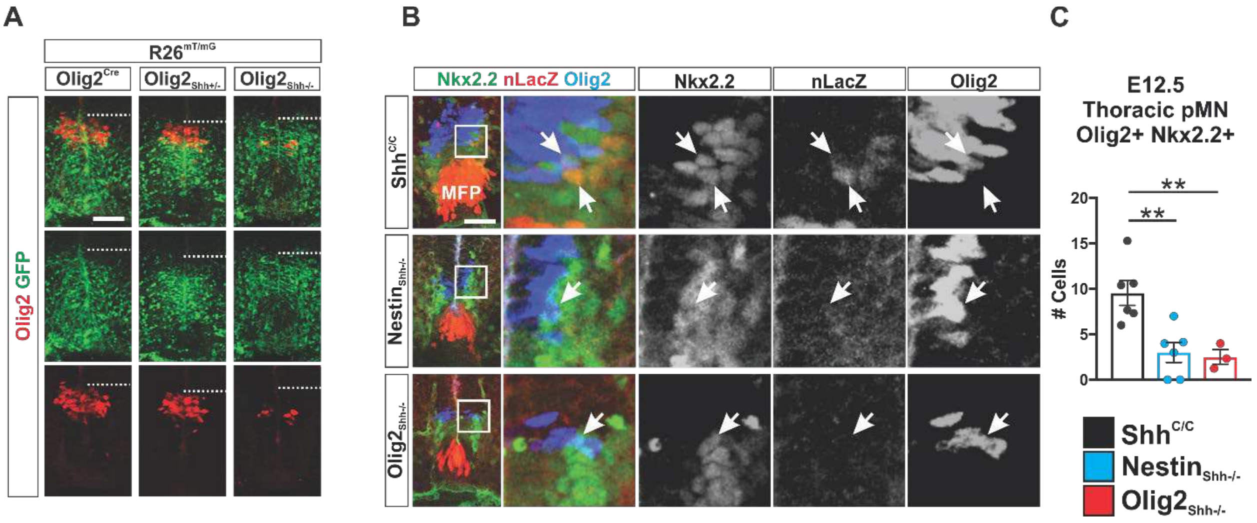

Supplementary Figure 6: Maintenance of Olig2+ cells in different pMN subdomains is dependent on Shh from different sources.

(**A**) E12.5 Lineage tracing reveals correct establishment of the pMN in $Olig2_{Shh}^{-/-}$ as indicated by dorsal boundary of R26mT/mG expression, however failure of maintenance of Olig2 in pMN as detected by immunolabeling. Scale bar, 50 µm.
(**B**) p* domain is found in contact with LFP* in $Shh^{C/C}$ controls but not in $Nestin_{Shh}^{-/-}$ and $Olig2_{Shh}^{-/-}$ as identified by immunolabeling of Olig2+ Nkx2.2 (p*) and nLacZ+ Nkx2.2+ (LFP*). Scale bar, 25 µm.
(**C**) Quantification of p* domain in $Shh^{C/C}$, $Nestin_{Shh}^{-/-}$, and $Olig2_{Shh}^{-/-}$. Means ± SEM are shown. $Shh^{C/C}$ n=6, $Nestin_{Shh}^{-/-}$ n=6, $Olig2_{Shh}^{-/-}$ n=3. One-way ANOVA, Dunnett's multiple comparison post hoc test. ∗∗p<0.01.

We lineage traced Olig2 cells in $Olig2_{Shh}^{-/-}$ mutants, $Olig2_{Shh}^{+/-}$ heterozygotes, and Olig2-Cre controls using the R26mT/mG reporter allele (**Fig. 5A**) from which myristilated GFP is expressed in all derivatives of Olig2 expressing cells and detected current Olig2 expression by immunohistochemistry at E12.5 (**supplemental Fig. S6A**). We observed GFP expression in the ventral spinal cord forming a dorsal boundary at a similar relative distance to the MFP in mutants and controls (dotted white line, **supplemental Fig. S6A**) but a decline in pMN_{Olig2}^{+} GFP^{+} double positive cells in mutants compared to controls. In particular, in the dorsal half of the pMN domain there was a complete absence of Olig2 expressing cells within the GFP labeled area in

mutants (**supplemental Fig. S6A**). These results suggested that the olig2+ cell population in the most dorsal part of the pMN domain is particular sensitive to reduced Shh signaling.

We find a ~3-fold reduction in the size of p* precursor domain in $Olig2_{Shh}^{-/-}$ compared to controls (**supplemental Fig. S6B and C**). The p* domain forms at the ventral border of the pMN domain and is marked by Olig2+/Nkx2.2+ cells (Agius et al., 2004). Interestingly, we found Nkx2.2+, Shh (nLacZ)+ LFP* cells in direct contact with Nkx2.2+ Olig2+ double positive cells of the p* domain in $Shh^{C/C}$ controls, highlighting a cyto-architectural arrangement that could underpin the disproportionate importance of LFP compared to MFP_{Shh} for the maintenance of the pMN_{Olig2}^{+} cell population at thoracic levels (**supplemental Fig. S6B and C**).

Supplementary Table 1: Reagents and Resources:

REAGENT	SOURCE	IDENTIFIER
**Antibodies**		
Chicken polyclonal anti beta Galactosidase	Abcam	Cat# ab9361, RRID:AB_307210
Goat polyclonal anti beta Galactosidase	Biogenesis	Cat# 4600-1409, RRID:AB_2314510
Goat polyclonal anti Choline acetyltransferase	Millipore	Cat# AB144P, RRID:AB_2079751
Rabbit polyclonal anti Olig2	Millipore	Cat#AB9610, RRID:AB_570666
Mouse monoclonal anti Nkx2.2	DSHB	Cat# 74.5A5, RRID:AB_531794
Rabbit polyclonal anti nNos	Immunostar	Cat# 24431, RRID:AB_572255
Mouse monoclonal Ankyrin G (AnkG) (463)	Santa Cruz	Cat# sc-12719, RRID:AB_626674
Mouse monoclonal Lim3 (Lhx3)	DSHB	Cat# 67.4E12, RRID:AB_2135805
Rabbit polyclonal Lim1	Gift Jessell Lab	N/A
Guinea Pig polyclonal Hb9	Gift Jessell Lab	N/A
Rabbit polyclonal FoxA2	Gift Jessell Lab	N/A
Rabbit polyclonal Pax6	Gift Jessell Lab	N/A
Rabbit polyclonal Dbx1	Gift Jessell Lab	N/A
Rabbit polyclonal Isl1/1	Gift Jessell Lab	N/A
Rabbit polyclonal anti Nkx2.2	Gift Jessell Lab	N/A
Donkey anti rabbit Alexa 488	Jackson ImmunoResearch	Cat# 711-545-152, RRID:AB_2313584
Donkey anti mouse Alexa 488	Jackson ImmunoResearch	Cat# 715-545-150, RRID:AB_2340846
Donkey anti guinea pig Alexa 488	Jackson ImmunoResearch	Cat# 706-545-148, RRID:AB_2340472

Donkey anti goat Alexa 488	Jackson ImmunoResearch	Cat# 705-545-147, RRID:AB_2336933
Donkey anti rabbit Cy3	Jackson ImmunoResearch	Cat# 711-165-152, RRID:AB_2307443
Donkey anti mouse Cy3	Jackson ImmunoResearch	Cat# 715-165-150, RRID:AB_2340813
Donkey anti guinea pig Cy3	Jackson ImmunoResearch	Cat# 706-165-148, RRID:AB_2340460
Donkey anti rat Cy3	Jackson ImmunoResearch	Cat# 712-165-153, RRID:AB_2340667
Donkey anti rabbit Alexa 594	Jackson ImmunoResearch	Cat# 711-585-152, RRID:AB_2340621
Donkey anti mouse Alexa 594	Jackson ImmunoResearch	Cat# 715-585-150, RRID:AB_2340854
Donkey anti guinea pig Alexa 594	Jackson ImmunoResearch	Cat# 706-585-148, RRID:AB_2340474
Donkey anti rat Alexa 594	Jackson ImmunoResearch	Cat# 712-585-153, RRID:AB_2340689
Donkey anti chicken Cy5	Jackson ImmunoResearch	Cat# 703-175-155, RRID:AB_2340365
Donkey anti goat Cy5	Jackson ImmunoResearch	Cat# 705-175-147, RRID:AB_2340415
Donkey anti mouse Cy5	Jackson ImmunoResearch	Cat# 715-175-150, RRID:AB_2340819
Donkey anti guinea pig Alexa Cy5	Jackson ImmunoResearch	Cat# 706-175-148, RRID:AB_2340462
**Chemicals**		
X-Gal	Roche	10745740001; CAS 7240-90-6
DAPI	Sigma Aldrich	CAS 28718-90-3
**Commercial Assays**		
Click-iT EdU	Thermofisher	C10340

**Mouse Strains**		
Mouse: Shh^{tm1Ahk}	Gonzalez-Reyes L, et al., 2012	MGI:5440762
Mouse: B6;129S6-$Chat^{tm1(cre)Lowl}$/J	The Jackson Laboratory	JAX: 006410
Mouse: B6.Cg-Tg(Nes-cre)1Kln/J	The Jackson Laboratory	JAX: 003771
Mouse: $Olig2^{tm1(cre)Tmj}$	Dessaud E, et al., 2007	MGI:3774124
Mouse: Gt(ROSA)$26Sor^{tm4(ACTB-tdTomato,-EGFP)Luo}$/J	The Jackson Laboratory	JAX: 007576

Supplementary Files

This is a list of supplementary files associated with this preprint. Click to download.

- nrreportingsummaryforShhscalesOPCnumbersStarikovetal.pdf
